# The Value of Magnetic Resonance Imaging Histograms in the Preoperative Differential Diagnosis of Endometrial Stromal Sarcoma and Degenerative Hysteromyoma

**DOI:** 10.3389/fsurg.2021.726067

**Published:** 2021-09-10

**Authors:** Xiao-Nan Zhang, Man Bai, Ke-Ran Ma, Yong Zhang, Cheng-Ru Song, Zan-Xia Zhang, Jing-Liang Cheng

**Affiliations:** Department of Magnetic Resonance, The First Affiliated Hospital of Zhengzhou University, Zhengzhou, China

**Keywords:** endometrial stromal sarcoma, degenerative hysteromyoma, magnetic resonance imaging, histogram analysis, preoperative differential diagnosis

## Abstract

**Objective:** The present study aimed to explore the application value of magnetic resonance imaging (MRI) histograms with multiple sequences in the preoperative differential diagnosis of endometrial stromal sarcoma (ESS) and degenerative hysteromyoma (DH).

**Methods:** The clinical and preoperative MRI data of 20 patients with pathologically confirmed ESS and 24 patients with pathologically confirmed DH were retrospectively analyzed, forming the two study groups. Mazda software was used to select the MRI layer with the largest tumor diameter in T2WI, the apparent diffusion coefficient (ADC), and enhanced T1WI (T_1_CE) images. The region of interest (ROI) was outlined for gray-scale histogram analysis. Nine parameters—the mean, variance, kurtosis, skewness, 1st percentile, 10th percentile, 50th percentile, 90th percentile, and 99th percentile—were obtained for intergroup analysis, and the receiver operating curves (ROCs) were plotted to analyze the differential diagnostic efficacy for each parameter.

**Results:** In the T2WI histogram, the differences between the two groups in seven of the parameters (mean, skewness, 1st percentile, 10th percentile, 50th percentile, 90th percentile, and 99th percentile) were statistically significant (*P* < 0.05). In the ADC histogram, the differences between the two groups in three of the parameters (skewness, 10th percentile, and 50th percentile) were statistically significant (*P* < 0.05). In the T_1_CE histogram, no significant differences were found between the two groups in any of the parameters (all *P* > 0.05). Of the nine parameters, the 50th percentile was found to have the best diagnostic efficacy. In the T2WI histogram, ROC curve analysis of the 50th percentile yielded the best area under the ROC curve (AUC; 0.742), sensitivity of 70%, and specificity of 83.3%. In the ADC histogram, ROC curve analysis of the 50th percentile yielded the best area under the ROC curve (AUC; 0.783), sensitivity of 81%, and specificity of 76.9%.

**Conclusion:** The parameters of the mean, 10th percentile and 50th percentile in the T2WI histogram have good diagnostic efficacy, providing new methods and ideas for clinical diagnosis.

## Introduction

Endometrial stromal sarcoma (ESS) is a rare gynecological tumor and the second most malignant mesenchymal tumor after uterine smooth-muscle sarcoma (1), accounting for 0.2–1% of uterine malignancies and <10% of uterine mesenchymal tumors (2). Endometrial mesenchymal tumors and related tumors can be categorized into different subtypes, including low-grade malignant ESS, highly malignant ESS, and undifferentiated uterine sarcoma (UUS) (3). In general, ESS manifests as a large polypoid mass in the endometrial cavity with varying degrees of myometrial infiltration, and distant metastases can occur at an early stage.

Uterine fibroids are the most common benign tumors of the female reproductive system. When the fibroids grow to a certain extent and degeneration occurs within the tumor (degenerative hysteromyoma, DH), the manifestation in conventional magnetic resonance imaging (MRI) is similar to that of ESS and, therefore, is easily misdiagnosed. Early differentiation between these two types of tumors is crucial in guiding treatment and the choice of surgical approach. For example, uterine fibroids can be treated with a range of approaches, including observation, hormonal therapy, uterine artery embolization, myomectomy, and simple hysterectomy (4), whereas ESS requires staged surgery, including total extrafascial hysterectomy with or without bilateral salpingo-oophorectomy (5), which minimizes the risk of abdominal spread.

Texture analysis is a form of radiomics that refers to the quantitative measurement of histograms and the distribution or relationship of the pixel intensities within a region of interest (ROI) on an image. It provides a more complex characterization of a lesion than traditional metrics by assessing the lesion's heterogeneity and reflecting the characteristic parameters in the overall lesion (6). The quantitative measurements this method generates can also provide useful information for the identification of tumors. Histograms have been shown to be valuable in the diagnosis of gastrointestinal tumors (7, 8), breast tumors (9), prostate tumors (10), and head and neck tumors (11). Some studies have used the histograms of the apparent diffusion coefficient (ADC) to differentiate the uterine sarcoma from endometrial cancer (12) and to diagnose the histological grading of endometrial cancer (13). However, few studies have used multiple MRI parameters for analysis and differential diagnosis.

The present study aimed to evaluate the role of histograms with multiple MRI sequences in the analysis and differential diagnosis of ESS and DH.

## Materials and Methods

### General Data

The data of patients meeting the following criteria from January 2016 to December 2020 were retrospectively analyzed.

Inclusion criteria: (1) patients with ESS or DH confirmed by surgical pathology; (2) patients who underwent routine MRI within 2 weeks before surgery, including axial T1WI, T2WI, diffusion-weighted imaging (DWI), ADC, and contrast-enhanced (CE) T1WI (T_1_CE); (3) patients with a single tumor with a maximum diameter ≥2.0cm or multiple tumors with a maximum diameter of the largest lesion ≥2.0cm (only the largest lesion was analyzed).

Exclusion criteria: (1) patients who had received other relevant treatment before the MRI examination; (2) patients with other uterine diseases; (3) patients with poor MR image quality that affected analysis.

A total of 44 patients were enrolled, including 20 with ESS (15 cases with low-grade ESS, five cases with high-grade ESS), and 24 with DH. The age range in the ESS group was 21–76 years (44.8 ± 15.01), the main symptom was irregular vaginal hemorrhage, and the maximum diameter of the lesion was 3.0–8.5 cm. The age range in the DH group was 22–77 years (42.6 ± 11.63), the lesion was mainly detected by physical examination, and the maximum diameter of the lesion was 2.5–10.5 cm. The details are shown in Table 1.

**Table 1:** The clinical characteristics in the patients.

		**Endometrial** **stromal** **sarcoma** **(*n* = 20)**	**Degenerative** **hysteromyoma** **(*n* = 24)**	***P*-value**
Age (year)		44.8 ± 15.01	42.6 ± 11.63	0.841
Diameter of the lesion (cm)		6.57 ± 4.12	6.19 ± 4.45	0.724
Irregular vaginal bleeding		11	2	0.001
Lower abdominal pain		5	3	0.284
Increased menstrual flow		5	4	0.495
Lower abdominal mass		2	2	0.848
Found by physical examination		2	12	0.005
Vaginal discharge		1	0	0.268
Site	Muscle layer	11	21	0.040
	Uterine cavity	7	3	
	Pelvic cavity	2	0	

The ethics committee of our hospital approved the study, and the requirement for informed consent was waived.

### MRI Examination

Conventional plain MRI, DWI, and enhanced scanning with a Siemens Skyra 3.0T superconducting MR scanner (Siemens, Germany) was conducted in all patients, with a body coil and a scan center located 2 cm above the pubic symphysis. Patients were instructed to lie in a supine position and maintain steady breathing. Conventional MRI scan sequences included the plain T1WI (transverse axial), T2WI (transverse and sagittal), DWI (transverse axial), and DWI images, with b-values of 0 and 800 s/mm^2^, respectively. ADC maps were automatically reconstructed after scanning. Enhancement scans were performed by a rapid (<10 s) bolus injection of gadopentetate dimeglumine (Gd-DTPA) via the elbow vein with a high-pressure syringe at a dose of 0.2 mmol/kg and a rate of 2–3 ml/s. One phase of plain scanning was conducted before injection, and 23 phases of uninterrupted repeat scanning were performed after injection. Delayed scanning was then performed. The sequences and parameters are shown in Table 2.

**Table 2:** Imaging protocol for MRI sequences.

	**T_**1**_WI**	**T**_**2**_ **WI**		**Contrast enhanced**		**DWI**
**Parameters**	**Axial**	**Axial**	**Sagittal**	**Axial**	**Sagittal**	**Axial**
Sequence	2-D TSE	2-D TSE	2-D TSE	VIBE	VIBE	EPI
Repetition time (ms)	450	2200	4020	6.16	6.24	4400
Echo tine (ms)	18	90	90	3	3.02	85
Field of view (mm^2^)	320 × 320	240 × 240	240 × 240	320 × 320	320 × 320	280 × 280
Matrix	256 × 256	258 × 384	258 × 384	240 × 320	240 × 320	110 × 128
Slice thickness/gap (mm)	5/1.0	5/1.0	5/1.0	2/1.0	2/1.0	5/1.0
Averages	2	1	2	2	2	2
Acquisition time (s)	93	113	136	56	57	57

*TSE, turbo spin-echo; VIBE, volume Interpolated body examination; EPI, echo-planar imaging*.

### Image Analysis

**Figure 1:**
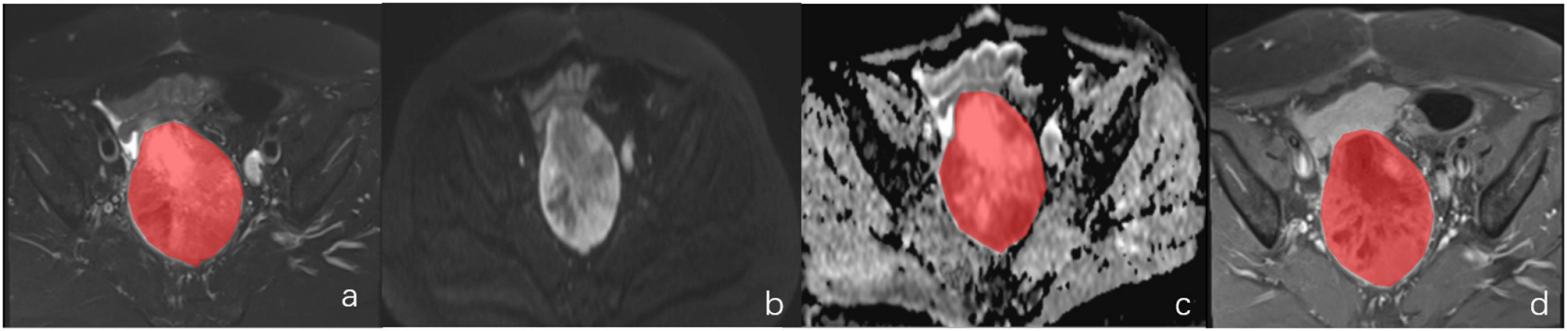
A female of 60 years with endometrial mesenchymal sarcoma. **(a–d)** represented the localization of the lesion on DWI and the ROI selection schematic for T2WI, ADC and T1CE, respectively.

**Figure 2:**
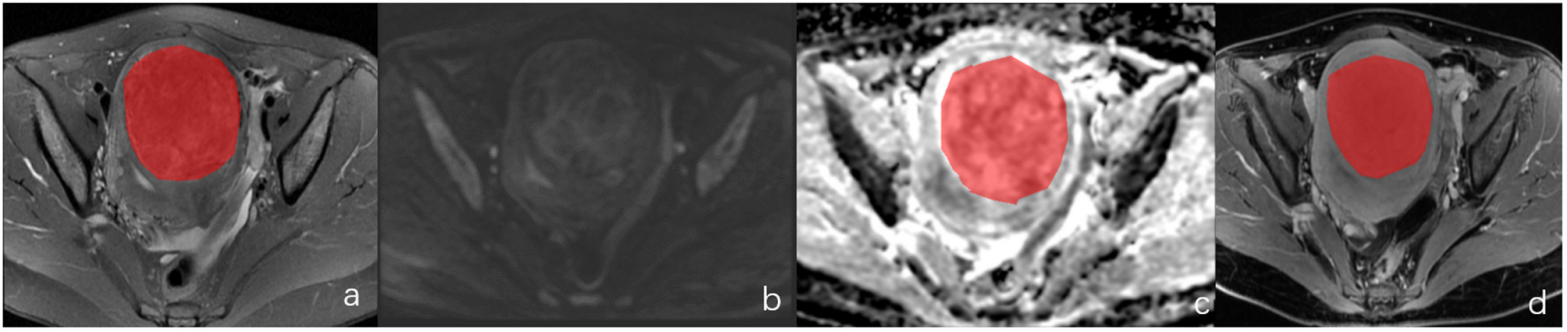
A female of 46 years with degenerative hysteromyoma. **(a–d)** represented the localization of the lesion on DWI and the ROI selection schematic for T2WI, ADC and T1CE, respectively.

**Figure 3:**
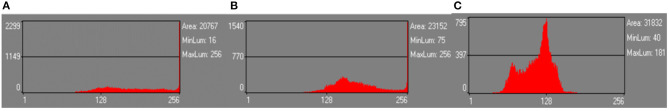
The same case as in Figure 1. **(A–C)** was the histograms of T2WI, ADC, and T1CE plots, respectively.

**Figure 4:**
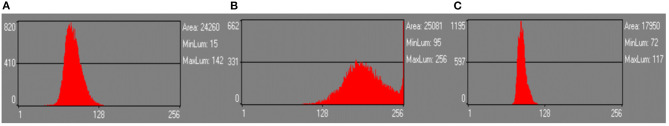
The same case as in Figure 2. **(A–C)** were the histograms of T2WI, ADC, and T1CE plots, respectively.

The MR images of all patients were exported from the PACS workstation and stored in.bmp format. For each patient, the T2WI, ADC, and T_1_CE images in the transaxial plane on the largest layer of the tumor were selected. The window width and position were adjusted so that all images were consistent. The selected transverse-plane T2WI, ADC, and T_1_CE images were analyzed using MaZda version 4.6 (Technical University of Lodz, Poland, http://www.eletel.p.lodz.pl/programy/mazda/). Before extraction of the texture feature, all images were normalized in the range of [μ – 3δ, μ + 3δ] (μ and δ were the average gray value and standard deviation, respectively) for the gray level to minimize the effects of contrast and luminance variations. The region of interest (ROI) was manually outlined along the edge of the lesion, the tumor area was filled with red, and the histogram was automatically generated by the software (see Figures 1–4). The horizontal coordinate in the histogram represented the different gray values within the ROI, and the vertical coordinate represented the frequency of occurrence of each gray value. The software automatically calculated the corresponding nine histogram parameters—the mean, variance, skewness, kurtosis, 1st percentile, 10th percentile, 50th percentile, 90th percentile, and 99th percentile. Two physicians with 5 and 3 years, respectively, of experience in MR imaging, then analyzed and measured the images using a double-blind method, and the average of the two was taken for analysis.

### Statistical Analysis

SPSS 21.0 statistical software was used for analysis, and the intra-class correlation coefficient (ICC) was utilized to evaluate the consistency of the results measured by the two observers. Data satisfying the normal distribution were expressed as mean ± standard deviation and tested using an independent sample *t*-test. Those not satisfying the normal distribution were expressed as median ± interquartile range and tested using the Mann–Whitney *U* test. *P* < 0.05 was considered statistically significant. The ROC curve and Youden index were used to evaluate the diagnostic efficacy of each histogram parameter.

## Results

### Consistency Test

The nine parameters (mean, variance, skewness, kurtosis, 1st percentile, 10th percentile, 50th percentile, 90th percentile, and 99th percentile) obtained from the T2WI, ADC, and T_1_CE images of the two types of tumors measured by the two observers were in good agreement, with the ICC ranging from 0.990 to 0.741 (*P* < 0.001). Therefore, the average value measured by the two observers was taken as the final evaluation index. The details are shown in Table 3.

**Table 3:** Consistency or correlation test for different parameter values between two observers.

**Parameters**	**Intra-group** **correlation** **coefficient (ICC)**	***P*-value**	**95% credibility interval**
**T**_**2**_ **WI degenerative hysteromyoma group (** ***n*** **=** **24)**			
Mean	0.990	*P* < 0.001	0.978–0.996
Variance	0.972	*P* < 0.001	0.936–0.998
Skewness	0.837	*P* < 0.001	0.66–0.926
Kurtosis	0.905	*P* < 0.001	0.794–0.958
1th percentile	0.949	*P* < 0.001	0.885–0.977
10th percentile	0.982	*P* < 0.001	0.960–0.992
50th percentile	0.992	*P* < 0.001	0.983–0.997
90th percentile	0.989	*P* < 0.001	0.975–0.995
99th percentile	0.986	*P* < 0.001	0.967–0.994
**ADC degenerative hysteromyoma group (** ***n*** **=** **24)**			
Mean	0.981	*P* < 0.001	0.955–0.922
Variance	0.908	*P* < 0.001	0.788–0.962
Skewness	0.924	*P* < 0.001	0.822–0.968
Kurtosis	0.924	*P* < 0.001	0.822–0.968
1thpercentile	0.941	*P* < 0.001	0.862–0.976
10th percentile	0.975	*P* < 0.001	0.939–0.990
50th percentile	0.978	*P* < 0.001	0.946–0.991
90th percentile	0.986	*P* < 0.001	0.965–0.994
99th percentile	0.894	*P* < 0.001	0.757–0.955
**T**_**1**_ **CE degenerative hysteromyoma group (** ***n*** **=** **24)**			
Mean	0.996	*P* < 0.001	0.989–0.998
Variance	0.990	*P* < 0.001	0.974–0.996
Skewness	0.941	*P* < 0.001	0.853–0.977
Kurtosis	0.911	*P* < 0.001	0.784–0.965
1th percentile	0.981	*P* < 0.001	0.952–0.993
10th percentile	0.992	*P* < 0.001	0.981–0.997
50th percentile	0.995	*P* < 0.001	0.897–0.998
90th percentile	0.995	*P* < 0.001	0.987–0.998
99th percentile	0.990	*P* < 0.001	0.974–0.996
**T**_**2**_ **WI endometrial stromal sarcoma (** ***n*** **=** **20)**			
Mean	0.987	*P* < 0.001	0.968–0.995
Variance	0.970	*P* < 0.001	0.927–0.988
Skewness	0.967	*P* < 0.001	0.918–0.987
Kurtosis	0.963	*P* < 0.001	0.908–0.985
1th percentile	0.983	*P* < 0.001	0.957–0.993
10th percentile	0.985	*P* < 0.001	0.961–0.994
50th percentile	0.985	*P* < 0.001	0.964–0.994
90th percentile	0.989	*P* < 0.001	0.973–0.996
99th percentile	0.978	*P* < 0.001	0.946–0.991
**ADC Endometrial stromal sarcoma (** ***n*** **=** **20)**			
Mean	0.984	*P* < 0.001	0.959–0.994
Variance	0.916	*P* < 0.001	0.791–0.968
Skewness	0.891	*P* < 0.001	0.733–0.958
Kurtosis	0.864	*P* < 0.001	0.673–0.947
1th percentile	0.908	*P* < 0.001	0.772–0.965
10th percentile	0.959	*P* < 0.001	0.895–0.985
50th percentile	0.984	*P* < 0.001	0.958–0.994
90th percentile	0.975	*P* < 0.001	0.935–0.991
99th percentile	0.871	*P* < 0.001	0.689–0.950
**T**_**1**_ **CE endometrial stromal sarcoma (** ***n*** **=** **20)**			
Mean	0.867	*P* < 0.001	0.680–0.948
Variance	0.848	*P* < 0.001	0.639–0.940
Skewness	0.901	*P* < 0.001	0.755–0.962
Kurtosis	0.741	*P* < 0.001	0.431–0.895
1th percentile	0.871	*P* < 0.001	0.690–0.950
10th percentile	0.891	*P* < 0.001	0.732–0.958
50th percentile	0.899	*P* < 0.001	0.750–0.961
90th percentile	0.808	*P* < 0.001	0.558–0.924
99th percentile	0.827	*P* < 0.001	0.596–0.932

### Histogram Parameter Analysis

The variance and kurtosis parameters in the T2WI, ADC, and T_1_CE histograms did not satisfy either the normal distribution or variance χ^2^ test, so the Mann–Whitney U test was used to compare the two groups for these parameters. The remaining parameters in the T2WI, ADC, and T_1_CE histograms (mean, skewness, 1st percentile, 10th percentile, 50th percentile, 90th percentile, and 99th percentile) all satisfied the normal distribution and homogeneity of variance, so an independent sample *t*-test was adopted for comparison between groups for these parameters.

The results showed that, of the nine gray-scale parameters obtained from T2WI histograms, seven (mean, skewness, 1st percentile, 10th percentile, 50th percentile, 90th percentile, and 99th percentile) had statistically significant differences between the two groups (*P* < 0.05). However, the differences between the two groups in the variance and kurtosis parameters were not statistically significant (*P* > 0.05). In the comparison of ADC histogram parameters, there was a statistically significant difference between the two groups in three of the parameters (skewness, 10th percentile, and 50th percentile) (all *P* < 0.05). There was no statistically significant difference between the two groups in six of the parameters (mean, variance, kurtosis, 1st percentile, 90th percentile, and 99th percentile) (all *P* > 0.05). None of the nine parameters in the T_1_CE histogram showed statistically significant differences between the two groups (*P* > 0.05).

The statistical results of the histogram parameters across the two groups are shown in Table 4.

**Table 4:** Comparison of histogram parameters between the endometrial mesenchymal sarcoma and degenerative hysteromyoma.

**Parameters**	**T**_**2**_ **WI**			
	**Endometrial stromal sarcoma**	**Degenerative hysteromyoma**	**t/*Z* value**	***P*-value**
Mean	138.235 ± 47.807	100.316 ± 36.483	2.983	0.005
Variance	491.869 ± 603.06^*^	288.640 ± 565.32^*^	1.391	0.164
Skewness	−0.402 ± 0.761	0.451 ± 0.323	−2.725	0.012
Kurtosis	0.331 ± 1.99^*^	0.162 ± 1.21^*^	0.966	0.334
1th percentile	83.808 ± 32.163	61.260 ± 23.140	2.699	0.010
10th percentile	108.970 ± 38.857	76.830 ± 29.360	3.123	0.003
50th percentile	138.554 ± 49.907	98.397 ± 36.559	3.076	0.004
90th percentile	167.273 ± 56.419	126.383 ± 45.115	2.672	0.011
99th percentile	187.231 ± 55.493	150.506 ± 52.113	2.251	0.030
**Parameters**	**ADC**			
Mean	124.497 ± 27.440	143.851 ± 32.284	1.841	0.075
Variance	1275.840 ± 980.095	778.197 ± 548.32^*^	1.179	0.249
Skewness	0.918 ± 0.866	0.328 ± 0.579	−2.422	0.021
Kurtosis	2.151 ± 3.111	1.055 ± 2.15^*^	0.438	0.678
1thpercentile	65.357 ± 22.724	80.810 ± 27.595	1.737	0.092
10th percentile	87.714 ± 23.711	111.476 ± 29.159	2.537	0.016
50th percentile	119.286 ± 27.592	142.048 ± 32.538	2.150	0.039
90th percentile	169.357 ± 43.805	179.857 ± 41.242	0.720	0.477
99th percentile	211.571 ± 30.686	213.333 ± 33.386	0.158	0.876
**Parameters**	**T**_**1**_ **CE**			
Mean	113.232 ± 34.692	126.847 ± 35.379	1.181	0.246
Variance	471.668 ± 804.03^*^	388.465 ± 834.45^*^	0.547	0.599
Skewness	−0.122 ± 0.824	−0.135 ± 0.546	−0.055	0.956
Kurtosis	0.492 ± 1.57^*^	0.940 ± 1.05^*^	1.732	0.086
1th percentile	60.667 ± 32.275	76.526 ± 28.941	1.575	0.124
10th percentile	81.944 ± 35.331	99.263 ± 31.296	1.580	0.123
50th percentile	113.000 ± 36.746	127.632 ± 36.687	1.212	0.234
90th percentile	143.500 ± 41.899	153.790 ± 43.508	0.732	0.469
99th percentile	165.556 ± 48.653	168.316 ± 43.909	0.181	0.857

* *indicated that the data did not satisfy the normal distribution and were expressed as median ± interquartile range; The remaining data satisfied the normal distribution and were expressed as mean ± standard deviation*.

### ROC Analysis of the Histogram Parameters

The ROC and Youden indexes were used to evaluate the differential diagnostic efficacy of the seven meaningful parameters in the T2WI histogram and the three meaningful parameters in the ADC histogram in ESS and DH, respectively. The area under the curve (AUC), optimal threshold, sensitivity, specificity, Youden index, and *P*-values are shown in Table 5.

**Table 5:** Diagnostic efficacy of the histogram parameters for endometrial mesenchymal sarcoma and degenerative hysteromyoma.

**Parameters**	**T**_**2**_ **WI**					
	**AUC**	**Threshold**	**Sensitivity (%)**	**Specificity (%)**	**Youden index**	**P value**
Mean	0.735	125.01	70.0	79.2	0.49	0.005
Skewness	0.25	0.97	10.0	95.8	0.06	0.012
1th percentile	0.715	84.1	55.0	87.5	0.43	0.010
10th percentile	0.740	108.58	55.0	91.7	0.47	0.003
50th percentile	0.742	124.10	70.0	83.3	0.53	0.004
90th percentile	0.714	155.46	70.0	79.2	0.49	0.011
99th percentile	0.690	173.15	70.0	70.8	0.41	0.030
**Parameters**	**ADC**					
Skewness	0.310	−0.111	90.5	23.1	0.14	0.021
10th percentile	0.731	102.00	76.2	84.6	0.61	0.016
50th percentile	0.738	122.50	81.0	76.9	0.58	0.039

**Figure 5:**
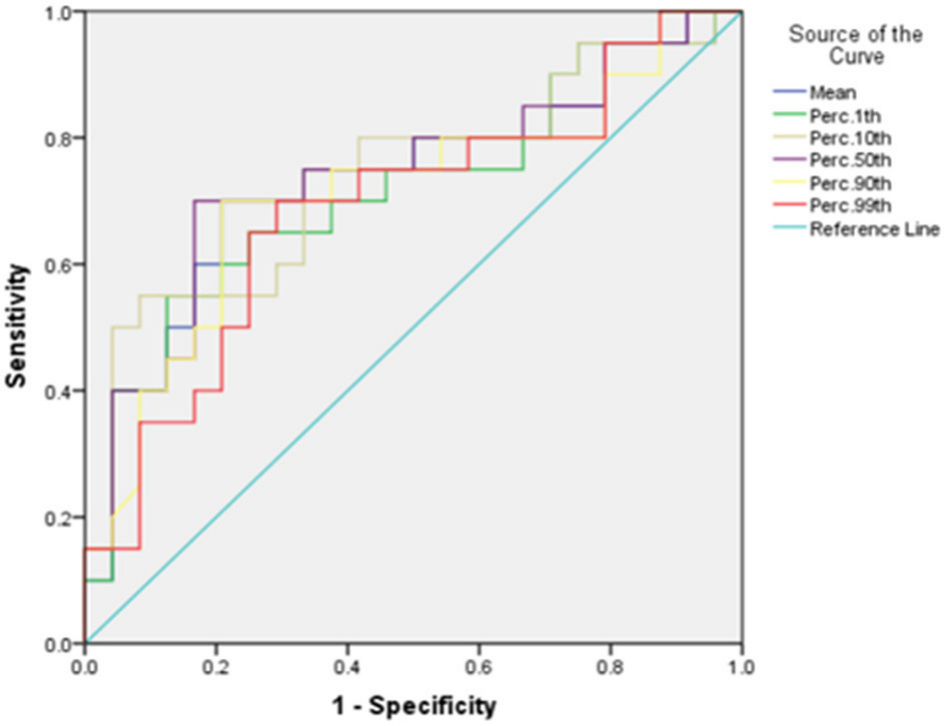
The ROC curves of the mean, 1st percentile, 10th percentile, 50th percentile, 90th percentile, and 99th percentile in T2WI histogram between the endometrial mesenchymal sarcoma group and degenerative hysteromyoma group.

**Figure 6:**
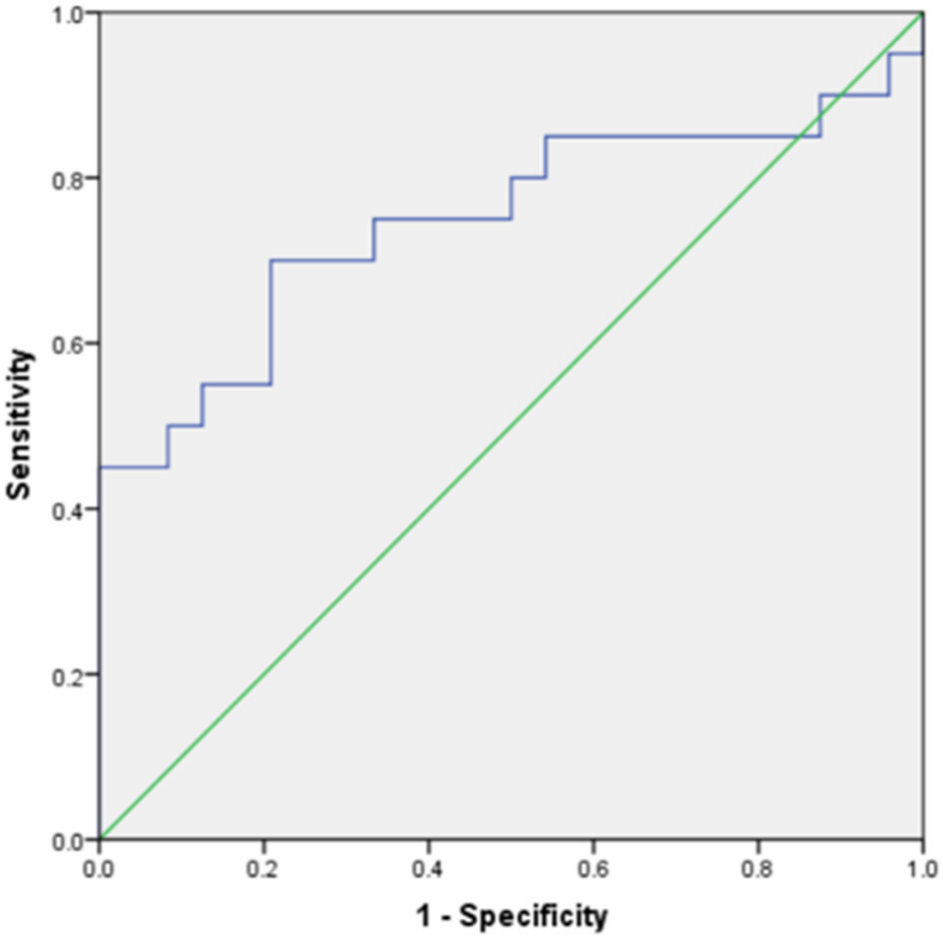
The ROC curve of skewness in the T2WI histogram between the endometrial mesenchymal sarcoma group and degenerative hysteromyoma group.

**Figure 7:**
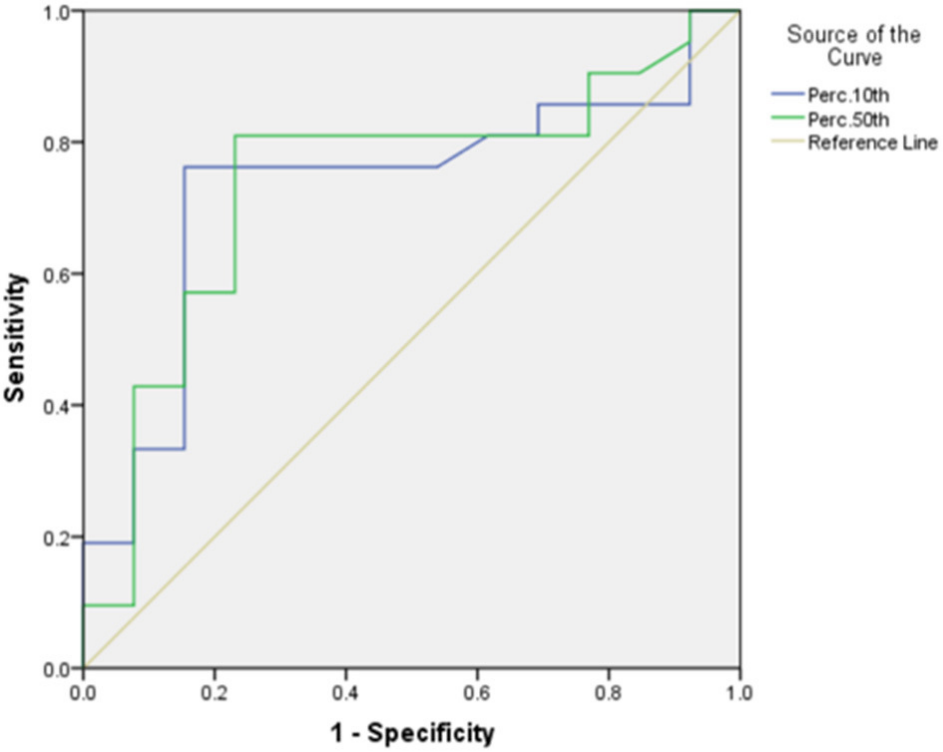
The ROC curves of the 10th percentile, 50th percentile in the ADC histogram between the endometrial mesenchymal sarcoma group and degenerative hysteromyoma group.

**Figure 8:**
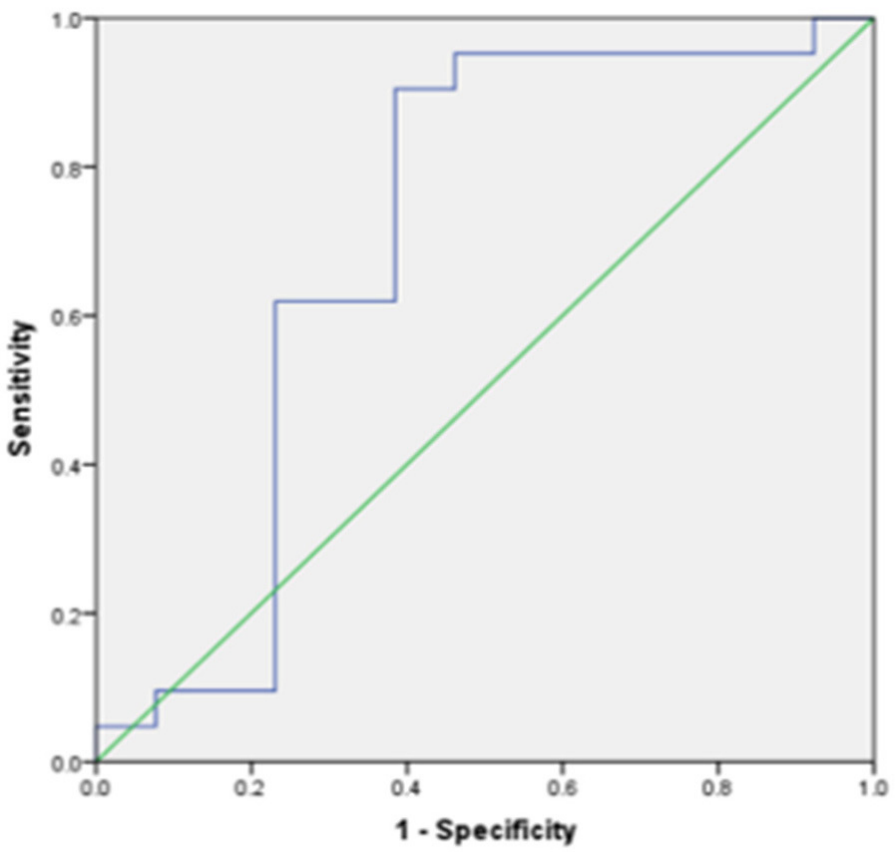
The ROC curve of skewness in the ADC histogram between the endometrial mesenchymal sarcoma group and degenerative hysteromyoma group.

Of the seven meaningful parameters in the T2WI histogram, five had an AUC > 0.7 (see Figures 5, 6), with the AUC for the 50th percentile being the largest (AUC = 0.742; *P* < 0.01) and having a sensitivity of 70% and a specificity of 83.3%. Of the three meaningful parameters in the ADC histogram, two had an AUC > 0.7 (see Figures 7, 8), with the AUC for the 50th percentile also being the largest (AUC = 0.738; *P* < 0.05) and having a sensitivity of 81% and a specificity of 76.9%.

## Discussion

ESS is a rare clinical malignancy of the female reproductive system originating from the endometrial mesenchymal cells, with a prevalence age of 40–60 years (14). It occurs mainly in the myometrium or endometrium and occasionally in extrauterine sites, such as the ovaries, peritoneum, and vagina (15). In the present study, ESS was located in the myometrium in 11 cases, in the uterine cavity in seven cases, and in the pelvis in two cases. Common symptoms of ESS are irregular vaginal bleeding, asymptomatic uterine enlargement, and lower abdominal or pelvic masses. On MRI, ESS appears as a heterogeneous high signal on T2WI, depending on the number of tumor components with cystic changes, hemorrhage, and necrosis. Previous studies have shown that the low signal bands within the infiltrated areas of the myometrium on T2WI, which correlate with the histopathologically preserved normal muscle bundles in the myometrium, are characteristic of ESS (16).

Uterine fibroids originate from the proliferation of uterine smooth-muscle cells and connective tissues and are most common in women of childbearing age. When the uterine fibroids degenerate, the lesion components are more complex, and the signal in T2WI is mostly heterogeneous. There is considerable overlap between ESS and DH regarding age and site of onset, clinical presentation, and imaging manifestations (17). This creates difficulties in the clinical differential diagnosis of the two conditions.

It has been previously reported that ADC values help to differentiate uterine sarcomas from DH (18), and the average ADC value in uterine sarcomas has been found to be significantly lower than in DH. In the present study, the differences in the average ADC values between the ESS and DH groups were statistically significant, and the average ADC value in the ESS group was smaller than that in the DH group, further demonstrating the importance of ADC values in the differential diagnosis between the two diseases. The present study also found that the differences in the average values between the ESS and DH groups were statistically significant in the T2WI histogram parameters and the parameters in the ESS group were slightly larger. This indicates that the lesions in the ESS group had higher signal intensity in the imaging, which might correlate with the fact that an ESS tumor resembles proliferative endometrial interstitial cell components and contains abundant mucus components, and the literature consistently reports this (19).

ESS has a rich blood supply and high microvessel density, showing obvious enhancement in the early stage and maintaining a high enhancement level in later stages. In contrast, enhancement in DH occurs later and is slightly lower (20). The differences in the average values between the two groups in the T_1_CE histogram were not statistically significant. However, the average value in the DH group was slightly higher than that in the ESS group, which was not consistent with previous literature. This may be because the T_1_CE delayed sequence scan time of 125 s after drug injection was adopted in the present study, meaning the delayed sequence failed to reflect the degree of enhancement at the early stage.

Previous studies have usually been limited to comparing the average values in ADC or T2WI, ignoring the heterogeneous features within a tumor. Gray-scale histogram analysis is a pixel distribution-based image analysis method that can extract the gray-scale intensity distribution of an ROI in a lesion in multi-parameter MR images for evaluation and obtain multiple histogram parameters. It can thereby objectively evaluate the heterogeneity of different types of tumors in a non-invasive and quantitative way. This technology can capture information about nuances that are invisible to the naked eye. With this image processing tool, the extracted histogram features can also be correlated with the biological behavior of tumors, which has clinical implications for tumor treatment and prognosis (21). Previous studies have mainly used the ADC histogram for tumor differentiation, but the histogram parameters in multiple MRI sequences for tumor analysis are less frequently studied. Currently, no relevant literature has been reviewed nationally or internationally on ESS identification using multiple MRI sequence histogram parameters.

In the present study, the histogram parameters in the T2WI, ADC, and T_1_CE histograms were analyzed. The results showed statistically significant differences in seven parameters in the T2WI histogram and three parameters in the ADC histogram, with the 10th percentile and 50th percentile showing better diagnostic efficacy. Of the seven T2WI histogram parameters, the 50th percentile had the best diagnostic efficacy (with an AUC of 0.742) and good sensitivity and specificity.

The percentile value describes the distribution of each gray value between the maximum and minimum values and is correlated with the heterogeneity of a tumor. The *n*th percentile means that *n*% of the data in the present column has a value less than or equal to this value (22). In the T2WI histogram, the 50th percentile in the ESS group was higher than that in the DH group, indicating that the 50th percentile voxel values in the ESS group were higher than those in the DH group. In the ADC histogram, the 50th percentile in the ESS group was lower than that in the DH group, indicating that the 50th percentile voxel values in the DH group were higher than those in the ESS group, and all the percentile values in the DH group were higher than those in the ESS group. This was correlated with the restricted diffusion of water molecules and lower ADC values caused by the active proliferation, increased cell density, tighter arrangement, and reduced extracellular space in the ESS group.

Kurtosis and skewness are parameters that describe the distribution of histogram curves and are commonly used indicators reflecting tumor heterogeneity (23). Kurtosis reflects the steepness of the distribution pattern of histogram gray-scale values—the larger the kurtosis, the greater the slope of distribution. In the present study, there was no statistically significant difference in kurtosis between the two groups of tumors on the T2WI, ADC, and T_1_CE histograms. The ESS group had higher kurtosis values on the T2WI and ADC histograms than the DH group, which might be attributable to the internal structure and greater heterogeneity of the various components of ESS than DH. Skewness reflects the asymmetry of the distribution of histogram gray-scale values—the larger the absolute value of the skewness, the greater the skewness of the distribution (24). In the present study, the differences between the two groups in skewness in the T2WI and ADC histograms were statistically significant. In the T2WI histogram, the skewness was negative in value, and the absolute value of skewness between the ESS and DH groups was not significantly different. This might be due to the complex composition of both types of tumors and the large heterogeneity within the tumors. However, the difference in skewness between the two groups in the ADC histogram has a better diagnostic value and high sensitivity.

The present study had some limitations. First, it was a retrospective analysis study, using a small sample without external validation; especially, the ESS group was not compared hierarchically. Further research will need to expand the sample size. Second, for the ROI, only the largest tumor layer was selected for histogram analysis, and the image texture information was not extracted comprehensively. Last, the correlation between the significance of each parameter and the biological mechanisms and clinical indicators of tumors was not sufficiently studied and needs to be investigated more thoroughly in future research. The research on the texture analysis of uterine tumors is still in the preliminary stage. Further studies are necessary before introducing radiomics features into the clinical workflow of uterine tumors.

## Conclusion

The findings in the present study suggest that the application of T2WI and ADC histogram analysis has clinical diagnostic value for the differentiation between ESS and DH. The mean, 10th percentile and 50th percentile parameters in the T2WI histogram have good diagnostic efficacy, providing new methods and ideas for clinical diagnosis.

## Data Availability Statement

The original contributions presented in the study are included in the article/supplementary material, further inquiries can be directed to the corresponding author/s.

## Ethics Statement

The studies involving human participants were reviewed and approved by ethics committee of The First Affiliated Hospital of Zhengzhou University. The patients/participants provided their written informed consent to participate in this study.

## Author Contributions

X-NZ, MB, and J-LC conceived the idea, conceptualized the study, and drafted and reviewed the manuscript. K-RM collected the data. YZ, C-RS, and Z-XZ analyzed the data. All authors read and approved the final draft.

## Conflict of Interest

The authors declare that the research was conducted in the absence of any commercial or financial relationships that could be construed as a potential conflict of interest.

## Publisher's Note

All claims expressed in this article are solely those of the authors and do not necessarily represent those of their affiliated organizations, or those of the publisher, the editors and the reviewers. Any product that may be evaluated in this article, or claim that may be made by its manufacturer, is not guaranteed or endorsed by the publisher.
